# Guidelines-Driven Educational Intervention Promotes Healthy Lifestyle Among Adolescents and Adults: A Serbian National Longitudinal Study

**DOI:** 10.3390/medicina55020039

**Published:** 2019-02-04

**Authors:** Višnja Đorđić, Predrag Božić, Ivana Milanović, Snežana Radisavljević, Maja Batez, Jagoda Jorga, Sergej M. Ostojić

**Affiliations:** 1 Faculty of Sport and Physical Education, University of Novi Sad, 21000 Novi Sad, Serbia; visnja@uns.ac.rs (V.Đ.); ivana.milanovic@fsfv.bg.ac.rs (I.M.); snezana.radisavljevic@fsfv.bg.ac.rs (S.R.); majabatezns@yahoo.com (M.B.); 2 National Institute of Sport and Sports Medicine, 11000 Belgrade, Serbia; predrag.bozic@rzsport.gov.rs; 3 School of Medicine, University of Belgrade, 11000 Belgrade, Serbia; jagoda.jorga@gmail.com

**Keywords:** healthy lifestyles, diet, physical activity, vegetable intake, food labels

## Abstract

*Background and objectives:* The effectiveness of short-term focused educational programs to change health behaviors across large populations seems to be poorly described so far. The main aim of the present study was to evaluate an age-specific 45-min educational program, designed in accordance with the current U.S. Department of Health and Human Services and U.S. Department of Agriculture dietary guidelines and physical activity (PA) guidelines, among adolescents and adults. *Materials and Methods:* We evaluated the health-promoting lifestyle habits by the Health-Promoting Lifestyle Profile (HPLP-II) at baseline and following 6–8 weeks post-education in a nationally representative sample of Serbian adolescents and adults (*n* = 3822). *Results:* The percentage of adolescents eating 3–5 servings of vegetables per day increased at follow-up (20.1% versus 23.1%, *p* = 0.001), with significantly more adolescents regularly reading food labels (from 12.2% at baseline to 14.2% at follow-up; *p* = 0.02). Taken together, mean HPLP-II scores in adolescents significantly improved for both diet (0.05 points; *p* < 0.0001) and PA (0.09 points; *p* < 0.0001), and for PA in adults (0.08 points; *p* < 0.0001). Hierarchical multiple regression analysis revealed that our model as a whole (including time of testing as a predictor variable, and age and gender as control variables) explained 3.0% of the variance in mean HPLP-II scores for diet (*p* = 0.942) and 3.0% for PA (*p* = 0.285) in adolescents, and 1.1% of the variance in HPLP-II scores for diet (*p* = 0.781) and 1.9% for PA (*p* = 0.075) in adults, respectively. *Conclusions:* It appears that a brief focused education can positively tackle unhealthy lifestyles in promoting good health in general population. Different modes of interactive communication used here appeared to strengthen participants’ capacities for lifestyle changes.

## 1. Introduction

Education about a proper diet and physical activity (PA) remains an important component of health promotion efforts [1], having in mind that people are becoming more sedentary and often eat unhealthy diets [2]. Global age-standardized prevalence of insufficient physical activity was 27.5% in 2016, with physical inactivity rates having increased over the past decade by 31.6% [3]. The unfavorable dietary patterns, characterized by a global increase in the consumption of processed foods, edible oils, and sugar-sweetened beverages, appears to tackle both low-income and high-income countries, with global dietary patterns based on unhealthy items worsened for 2.5 points from 1990 to 2010 [4]. As a consequence, the prevalence of overweight and obesity have increased among people of all ages to the highest rates ever documented, with global obesity prevalence predicted to reach 18% in men and surpass 21% in women by 2025 [5]. The economic burden of unhealthy behaviors balloons, with physical inactivity and poor nutrition persisting as major contributors to disease and death from chronic diseases [6,7,8]. For example, approximately 16.0 million (1.0%) disability adjusted life years (DALYs, a measure of the potential life lost due to premature mortality and the years of productive life lost due to disability) and 1.7 million (2.8%) of deaths worldwide are attributable to low fruit and vegetable consumption [9], while 3.2 million deaths each year are attributable to insufficient physical activity [10]. To tackle this, leading health authorities continuously develop and update global guidelines on healthy lifestyles to help general population embrace the knowledge, skills, and attitudes necessary to develop a healthful diet and be physically active [11,12,13,14,15,16,17]. Typically, these guidelines encourage people of all ages to regularly participate in physical activities that are appropriate for their age (e.g., at least 150 min a week of moderate-intensity activity in adults, or 60 min or more of physical activity daily in children and adolescents) [11], and consume a healthy diet across the lifespan by shifting to healthier food choices (such as nuts, whole fruits, and vegetables) while limiting added sugars, saturated fats, and sodium, in aim to prevent malnutrition and chronic diseases [15]. However, the effectiveness of guidelines-driven educational programs to change health behaviors across large populations seems to be insufficiently described so far.

This particularly applies to short-term educational interventions that tackle the adult population, while most previous studies evaluated the impact of medium- or long-term family or community interventions on heath behaviors in normal-weight and overweight school children and adolescents. For instance, participation in a one-year community-based, family-centered behavioral intervention appeared to have a positive influence on the physical activity, sedentary, and healthy eating behaviors of overweight and obese adolescents in Australia and New Zealand [18]. A multi-component lifestyle educational program was practical and effective in improving health behaviors and obesity-related knowledge for 1108 children in China [19]. A 20-month school-based health promotion intervention has a positive effect on vegetable consumption and psychological outcomes among 2119 secondary school adolescents from Peru [20]. Seven-month, intensive, school-wide intervention decreased weekday television and videogame time in 3592 children in Brazil [21]. Although low-powered, a community-based healthy lifestyle program for overweight/obese fathers (*n* = 93) and their primary school-aged children (*n* = 132) demonstrated improved health outcomes and behaviors after a 7-week intervention [22], thus providing an argument for applying time-effective programs across different age groups. Therefore, the main aim of the present study was to evaluate an age-specific short-term educational program, designed in accordance with the current U.S. Department of Health and Human Services (USDHHS) and U.S. Department of Agriculture (USDA) dietary and PA guidelines, in a nationally representative sample of Serbian adolescents and adults. This study thus provided rather novel information about the practical value of such time-efficient educational intervention carried out across the largest cohort representing the general population of one country to date, as far as known by the authors. 

## 2. Materials and Methods

### 2.1. The National Diet and Physical Activity for Health Initiative (DiPAH)

The Effectiveness of Targeted Educational Intervention on Healthy Lifestyle Behaviors in Serbian Population (TEDI-HL) (see below) is an extension sub-study of the Diet and Physical Activity for Health Initiative (DiPAH). The DiPAH was started in 2008 as a long-term, nationally-recognized health study that is focused on strategies for chronic disease prevention of children and adults in Serbian population. The DiPAH population comprises of Serbian children (aged 6.0 years and above) and adults from 24 official administrative divisions in Serbia (out of 29), who were invited by general media and specialized e-portals to take part in this initiative, agreed to voluntarily participate, and were evaluated (at least once) in the mobile examination unit for disease history, general health, dietary habits, physical activity, and/or health-related physical fitness. Prior to action, we undertook four rounds of focus workshops to balance recruitment for demographic, socioeconomic, age, and gender differences. Field examinations were organized each spring and fall, with six mobile groups testing ~100 participants per day. Population-based estimates of disease prevalence from DiPAH cohort were published through NCD-RisC collaboration network (www.ncdrisc.org) The TEDI-HL was set up to follow up subsamples of adolescents and adults initially enrolled through DiPAH database. 

### 2.2. Study Participants

Participants were selected to join the TEDI-HL on the basis of the following selection criteria: (a) previously participated in DiPAH trials; (b) not previously enrolled in a health education program, (c) had no medical conditions that would limit the successful completion of the protocol, and (d) had a current residence in Serbia. Age ranges for eligible participants were between 10.0 and 18.9 years for adolescents, and above 19.0 years of age for adults, as of October 2016. Of 11,069 DiPAH subjects initially considered, 3278 adolescents (age 13.2 ± 1.9 years, 49.5% girls) and 544 adults (age 52.2 ± 18.2 years, 58.5% women) were eligible and consented to participate in the current study (*n* = 3822). The study was conducted according to the guidelines laid down in the Declaration of Helsinki, and all procedures were approved by the local IRB at the University of Novi Sad (Ref. No. 15848/2016; approved at 1 October 2016). All adult participants provided informed consent before participating in the study; parents or other legally authorized representatives dispensed permission for minors to be included in the study. 

### 2.3. Study Design

The TEDI-HL is a longitudinal study designed to identify the effectiveness of a single 45-min session of focused education, designed in accordance with the current USDHHS/USDA dietary and PA guidelines, on health-promoting lifestyle habits in a nationally-representative sample of Serbian adolescents and adults. From October 2016 to May 2017, all participants (*n* = 3822) were visited in their schools, workplaces, or retirement homes by trained research staff who administered a Health-Promoting Lifestyle Profile (HPLP-II) questionnaire [23] and a Short-Form Health Survey (SF-12) [24] at baseline and at 8-week follow-up. The HPLP-II questionnaire is a 52-item self-report of health-promoting lifestyle habits in six domains, with PA and diet subscale scores separately analyzed in this study. Participants indicated how often they engage in certain health-promoting behavior on a four-point Likert scale ranging from 1 (never) to 4 (routinely). Mean scores were calculated for both subscales, with higher scores indicating more frequent health-promoting behavior. Diet and PA subscales have adequate internal consistency (Cronbach’s alpha coefficients were 0.81 and 0.73, respectively). SF-12 is a multipurpose, generic 12-item questionnaire of health status, developed as a shorter version of SF-36 [25]. Physical and mental health summary scores (PCS-12 and MCS-12) are yielded by standardization and norm-based scoring algorithm provided by the developers [24]; the procedure allows for cross-cultural comparison. The TEDI-HL was registered at clinicaltrials.gov as NCT02999425.

### 2.4. Educational Intervention

At the baseline visit, immediately after the initial HPLP-II and SF-12 assessment, participants attended a single 45-min educational session with a trained instructor, to receive written, verbal, and visual information about healthy diet and PA; adolescents received an instruction adjusted to their age. A session started with a general introduction about guidelines (~5 min), followed by 30-min oral and visual presentation of diet and PA advice using PowerPoint slides (version 16.16.6 for Mac, Microsoft Corp., Redmond, WA, USA), and finalized with a discussion/counseling forum (~10 min) aimed to answer any individual queries in each focus group. Group sizes varied from 15 to 30 participants, and age-specific materials (both printed and video) were developed through technical committee meetings. The applied educational intervention belongs to a knowledge, attitudes, practices model which strives to enhance positive health behavior choices and prevent negative ones [1]. The educational session provided unbiased information on healthy diet and PA in accordance with USDHHS/USDA dietary and physical activity guidelines [11,15]. Interactive educational strategies, including brainstorming, problem solving, and discussion, were implemented in order to facilitate active participation. In addition, a leaflet containing key health messages and infographics was provided to each participant. Both adolescents and adults were required to restrict from enrolling in other health education programs throughout the study. 

### 2.5. Statistical Analyses

All data were analyzed using IBM SPSS Statistics for Mac 21.0 software (SPSS Inc., Chicago, IL, USA). Descriptive statistics was used for general characteristics. Comparisons were made between the baseline data and follow-up data to identify the effects of the educational intervention program by paired *t*-test (for nominal data) and by chi-squared test (for proportions). In addition, a multiple linear regression model with a stepwise method (adjusted for age and gender) was employed to examine the association between an independent variable (time of testing) and a single continuous dependent variable (mean HPLP-II scores for diet and PA). A 2-tailed *p*-value < 0.05 was considered significant.

## 3. Results

Of 3822 participants initially evaluated, 94.7% completed post-intervention assessment at the follow-up. Changes in specific lifestyle habits related to diet in adolescents and adults are presented in Table 1. It appears that significantly higher number of both adolescents and adults reported regular use of low-fat foods at follow-up versus baseline (*p* < 0.05). The percentage of adolescents eating 3–5 servings of vegetables per day increased at follow-up (20.1% versus 23.1%, *p* = 0.001), with significantly more adolescents regularly reading food labels (from 12.2% at the baseline to 14.2% at the follow-up; *p* = 0.02). In addition, the percentage of adolescents that regularly eats breakfast dropped throughout the study from 78.2% at the baseline to 73.8% at the follow-up (*p* < 0.001). Other lifestyle habits related to diet were not affected by the intervention.

**Figure 1:**
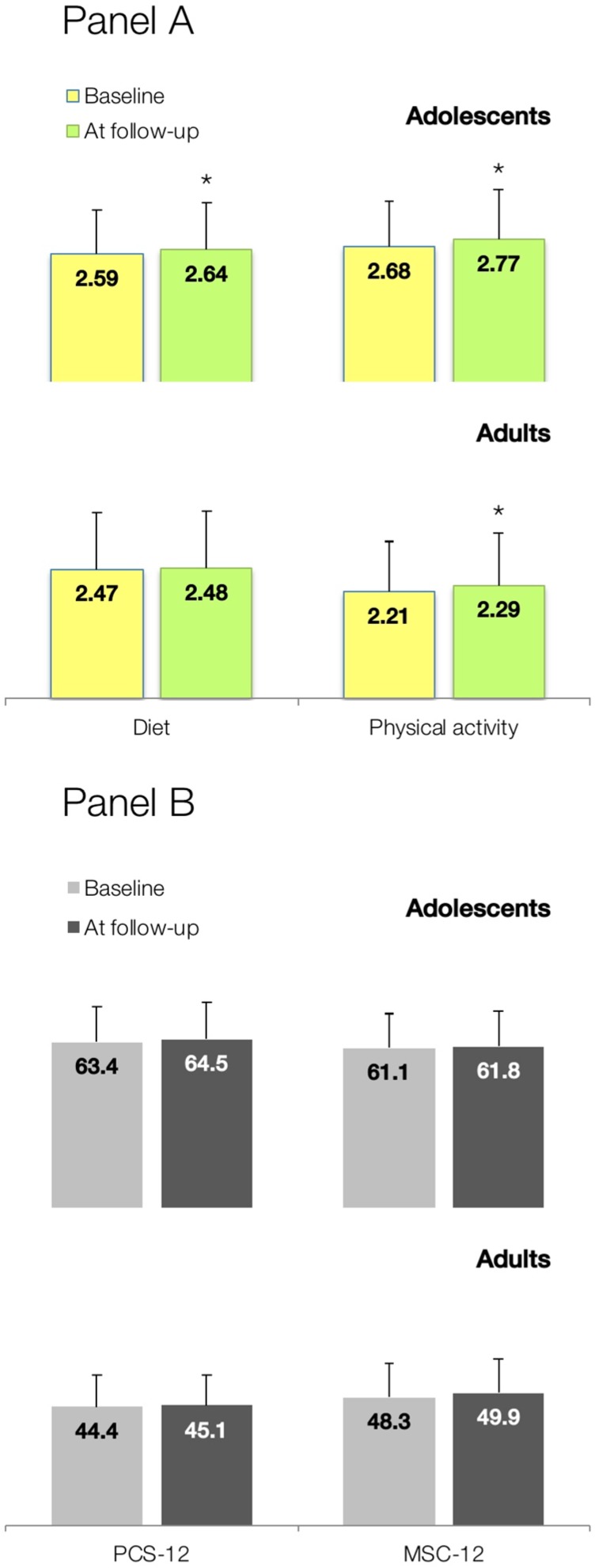
Changes in mean health-promoting lifestyle habits scores for diet and physical activity (Panel **A**), and Short-Form Health Survey (SF-12) physical (PSC-12) and mental health (MSC-12) summary measures (Panel **B**) after a focused educational session in a representative sample of Serbian adolescents and adults (*n* = 3822). Values are shown as mean scores ± SD. Asterisk (*) indicates significant changes at *p* < 0.05 between scores at baseline and follow-up.

PA lifestyle profiles are depicted in Table 2. A significant increase in the number of adolescents that exercise vigorously at least three times a week, take part in light to moderate PA, and regularly do stretching were found at the follow-up (*p* < 0.05). However, fewer adolescents were physically active during usual daily activities, and followed a planned exercise program at the follow-up (*p* < 0.05). Adults appeared to take part in leisure-time PAs more often after an educational intervention, routinely do stretching exercise at least 3 times per week, and more regularly check their pulse when exercising comparing to baseline (*p* < 0.05). Taken together, mean HPLP-II scores significantly improved for both diet (0.05 points, 95% CI from 0.03 to 0.07; *p* < 0.0001) and PA (0.09 points, 95% CI from 0.06 to 0.12; *p* < 0.0001) in the adolescent population, and for PA in adults (0.08 points, 95% CI from 0.05 to 0.11; *p* < 0.0001) (Figure 1). SF-12 physical and mental health summary scores tended to increase after the intervention in both adolescents and adults, yet not reaching statistical significance (*p* > 0.05).

Hierarchical multiple regression analysis revealed that our model as a whole (including time of testing as a predictor variable, and age and gender as control variables) explained 3.0% of the variance in mean HPLP-II scores for diet (*R* = 0.17; standard error of estimate = 0.47; *p* = 0.942) and 3.0% for PA (*R* = 0.18; standard error of estimate = 0.60; *p* = 0.285) in adolescents, and 1.1% of the variance in HPLP-II scores for diet (*R* = 0.12; standard error of estimate = 0.44; *p* = 0.781) and 1.9% for PA (*R* = 0.15; standard error of estimate = 0.65; *p* = 0.075) in adults, respectively. Age makes unique statistically significant contribution to our model of changes in health-promoting lifestyle habits for diet and for physical activity, while gender made no significant contribution to the model neither for diet or PA in adults, or for diet in adolescents (Table 3). However, gender made a unique significant contribution to our model for PA in adolescents.

## 4. Discussion

In this nationally-representative intervention trial, we found that an age-specific single 45-min interactive educational intervention, designed in accordance with USDHHS/USDA dietary and PA guidelines, turned out to be effective in terms of improving health-promoting lifestyle habits in Serbian adolescents and adults. Significantly higher scores for mean HPLP-II scores at 8-week follow-up perhaps reflected a positive change induced by a brief intervention that can tackle unhealthy behaviors and promote good health in general population. We also demonstrated here that age had a significant association with changes in HPLP-II scores for both diet and PA in our cohort of adolescents and adults, while gender had rather irrelevant contribution in predicting health-profile changes. Age seemed to be negatively associated with alterations in mean HPLP-II scores after education in adolescents, likely suggesting younger age of this population as more susceptible to change. On the other hand, a positive correlation found among adults perhaps suggest that participants with advanced age in this subsample are prone to change to a greater extent. Although comparatively short, the intervention was delivered in schools, workplaces and retirement homes across Serbia, which are a promising setting for implementation of health education programs since they cater for large proportion of adolescent and adult population.

Several recent studies evaluated the effects of short-term education on health-promoting lifestyle habits, using HPLP II scores as primary outcomes, in many different settings from workplace and hospital, to retirement homes and classroom environments. Eight sessions of 45–60 min face-to-face educational intervention, held weekly in two sessions over a period of 4 weeks, by PowerPoint lecture, discussion, and the provision of leaflets, improved HPLP II scores in 72 retired individuals [26]. Two-to-three months of education improved mean HPLP II scores for PA and nutrition in 86 patients with hypertension [27], and 80 patients who had undergone coronary artery bypass surgery [28]. An 8-h program significantly increased overall health promoting behaviors in 100 hospital registered nurses [29]. Health-promoting lifestyle profiles were also improved after teacher-led educational intervention among public high-school students in Boston [30]. Taken together, the overall mean score for the HPLP II across studies was ~2.5 points at the baseline, with a specific educational activity improve scores at post-intervention regardless of the duration of an education. Previous studies were often small and low-powered, presented only mean scores for HPLP II, and also omitted to recruit (and contrast) participants from different age groups. In our study with 3822 participants, we found that adolescents reported rather healthier nutrition and PA habits than adults, both at the baseline and at follow-up. At the same time, adolescents had the higher mean score in PA than in diet, while adult group scored the other way around, which perhaps reflects the age-related PA decrease [31]. Since a mean of 2.50 can be considered a positive response, adolescents’ mean scores ranging from 2.59 (diet at the baseline) to 2.77 (PA at the follow-up) in our study seems to reflect acceptable level of health-promoting lifestyle habits in Serbian adolescents, with scores were in accordance with previous research [32]. However, it is not the case in adult population, where mean scores for Diet and PA ranged from 2.21 (PA at the baseline) to 2.48 (diet at the follow-up).

For individual health-promoting lifestyle behaviors before the intervention, the most unfavorable dietary habit in adolescents referred to fat consumption, with less than 8% routinely choosing a diet low in fat. Around one in ten adolescents ate the recommended amount of bread, cereal, rice, and pasta on a regular basis, while no more than 12% regularly read food labels. Other health-promoting lifestyle habits were also underrepresented in adolescent population, including limited use of sugars and sweets, recommended vegetable and protein food consumption, as well as consumption of dairy products and fruit. At the same time, health-promoting lifestyle habits concerning fruit and vegetable consumption were present to a strikingly lesser extent in the adolescent population, reaching in some cases just half of the percentage of adults reporting the same behavior as a regular one. This is in line with previous studies describing poor dietary habits among young people, particularly for fruit and vegetables intake [33], a practice that may negatively impact both school performance and health [34]. After the intervention, some adolescents’ dietary behaviors improved-consumption of food with low fat content, vegetable consumption, and food label reading. This is in line with previous trials confirming an education-generated potential for behavioral change in this population [18,19,20,21,22], even after a short-term intervention employed in our study. However, in adult population the intervention positively influenced only consumption of low-fat food. This is perhaps due to higher resistance to change behaviors related to health in adults [35], and/or due to somewhat limited efficacy of short-term educational interventions in this age group [36]. Age was found to be a strong predictor of HPLP II change for dietary habits in our cohort, thus justifying a need for age-specific strategies to tackle unhealthy behaviors in large populations. 

For the PA domain, adolescents and adults reported several unfavorable habits both before and after the educational intervention. For instance, 39.4% of adolescents and 55.9% of adults regularly took part in light-to-moderate PA, an activity that has many health benefits in any age group [7]. In addition, only 7.2% of the youth population and 17.2% of the adult population routinely checked their pulse when exercising, a method that provides an effective monitoring of health-promoting physical activity [37]. Comparable to intervention’s effects on dietary habits, the follow-up results suggest that a single education session may induce changes in health-enhancing PA behaviors. A significantly higher percentage of both the adolescent and adult population reported regular stretching activities post intervention. In addition, adolescents improved in terms of weekly vigorous and moderately vigorous PA engagement and in attaining their target heart rate when exercising, while in the adult population improvements were identified in leisure-time PA and pulse control during exercise. However, it seems that a few PA behaviors are impervious to change or even got worse at the post intervention. This probably illustrates a limited capacity of short educational intervention employed here to produce a desirable change in PA habits.

It seems that adolescents responded better to the educational intervention than the adults, although the education was age-tailored. There might be a few possible explanations for this. The adult group consisted of a fairly large proportion of elderly people living in retirement-homes, which provide main meals for their residents, thus limiting room for a change in dietary behaviors. Besides, elderly people might be more reluctant to change in general [38], with institutionalized older adults being passive in particular [39]. The normal aging process is related to a gradual decline in some cognitive abilities (e.g., memory, processing speed, conceptual reasoning, attention), which may affect the learning ability in this population [40]. They might experience a lack of social support needed for effective lifestyle change; actually, older adults receive less health-related social control in comparison to younger and middle-aged adults [41]. It has been proved that social networks facilitate PA and reduce the odds of obesity for 55-year-old-plus adults [42]. As for the Serbian population, a recent study showed that Serbia scores 27th out of 29 European countries in terms of PA, with only 3.6% of older people being engaged in regular PA [43]. The sedentary lifestyle of elderly participants included in the adult sample might have affected the results of our study. In addition, when explaining differences between adolescents and adults, it has to be taken into account that eagerness to initiate the health behavior changes and short-term outcomes seem to be in favor of young people, yet maintaining the change could be more challenging for them [44].

The main focus of an effective health education is the empowerment of individuals to make healthy choices in everyday life. Gaining knowledge and motivation through education, alongside the environmental support, enables people to change unhealthy lifestyle habits. This might be of special importance in transition countries like Serbia, where modifiable behaviors related to unhealthy diet and physical inactivity might worsen with overall development. A recent analysis of the Serbian healthcare system recommends a shift from curative to preventive medicine, since promotion of a healthy lifestyle decreases healthcare costs in the mid- to long-term [45]. At the same time, quality data on time- and cost-effective health education programs in the region is still lacking. 

### Study Limitations

Several limitations must be considered when study findings are interpreted. Although initial results of our intervention in changing diet and physical activity health profiles are encouraging and corroborate previous findings [18,19,20], especially in adolescents, this kind of intervention is perhaps too short and should be accompanied by environmental support, in order to provide sustained change in health enhancing lifestyle habits. We recruited here over 3800 participants, yet the length of the study (6–8 weeks) remains an important weakness of the current trial. Also, we have not enrolled children aged <10.0 years, a population well recognized for its high sensitivity to health promotion interventions [33,46]. Therefore, it remains unknown whether healthy lifestyle profiles of pre-adolescents change after short-term health education. Besides, large cohorts such as TEDI-HL should be followed more frequently to get the measure of knowledge retention, which might determine sustainable health benefits [41]. The downsides of the one-group pretest-posttest design employed in our trial, could be overcome by adding more design elements in future studies, such as the control group that receives no organized educational intervention. In addition, controlling for other possible confounding factors in the course of focused health education (such as advertising or lay media) might help us to leverage a real-life value of such an intervention. Finally, further studies should also monitor the long-term changes in health status and objectively measured biomarkers of large cohorts that receive educational intervention promoting healthy lifestyles, particularly for sub-populations suffering from chronic diseases (e.g., hypertension, diabetes, obesity). Although with limitations, this trail described a broad population, and invites for collection of additional information from other resources to address research gaps.

## 5. Conclusions

This study was the first to evaluate the effects of a brief educational intervention on dietary and physical activity behavior in a representative sample of Serbian adolescents and adults. The study provided promising results concerning positive impact of the intervention on health-enhancing lifestyle habits, particularly in a physical activity domain and in favor of the adolescent group. It is of a great importance to target young people by educational interventions, because health behaviors can be tracked down from adolescence into adulthood, thus affecting health throughout the life course [47]. However, tackling unhealthy lifestyle of adults might need another approach in public health initiatives.

## Figures and Tables

**Table 1:** Self-report of health-promoting lifestyle habits for diet during the study. Values are reported as percentages of participants who routinely comply with the guidelines.

	Adolescents		*p*	Adults		*p*
	Baseline (*n* = 3278)	At Follow Up (*n* = 3105)		Baseline (*n* = 544)	At Follow Up (*n* = 515)	
Choose a diet low in fat, saturate fat, and cholesterol	7.7	9.2	0.03	35.6	41.8	0.04
Limit use of sugars and food containing sugar (sweets)	16.7	16.9	0.83	40.8	46.3	0.07
Eat 6–11 servings of bread, cereal, rice, and pasta each day	10.1	11.0	0.24	19.6	22.9	0.19
Eat 2–4 servings of fruit each day	34.7	33.9	0.50	42.6	43.6	0.74
Eat 3–5 servings of vegetables each day	20.1	23.1	0.00	39.9	40.5	0.84
Eat 2–3 servings of milk, yogurt, or cheese each day	34.1	34.9	0.50	46.7	46.8	0.97
Eat only 2–3 servings from the protein group each day	26.6	26.1	0.65	47.6	42.1	0.07
Read labels to identify nutrients, fats, sodium content in packaged food	12.2	14.2	0.02	42.0	40.0	0.51
Eat breakfast	78.2	73.8	0.00	87.3	83.0	0.05

**Table 2:** Self-report of health-promoting lifestyle habits for physical activity during the study. Values are reported as percentages of participants who routinely comply with the guidelines.

	Adolescents		*p*	Adults		*p*
	Baseline (*n* = 3278)	At Follow Up (*n* = 3105)		Baseline (*n* = 544)	At Follow Up (*n* = 515)	
Follow a planned exercise program	44.8	41.2	0.00	27.2	31.4	0.13
Exercise vigorously for 20 or more minutes at least three times a week	43.5	47.3	0.00	43.7	43.4	0.92
Take part in light to moderate physical activity	39.4	42.5	0.01	55.9	57.7	0.56
Take part in leisure-time (recreational) physical activities	29.1	31.4	0.05	30.8	36.8	0.04
Do stretching exercises at least 3 times per week	40.9	43.4	0.04	28.6	35.5	0.02
Get exercise during usual daily activities	72.9	69.3	0.00	63.3	58.8	0.13
Check my pulse rate when exercising	7.2	7.4	0.76	17.2	22.3	0.04
Reach my target heart rate when exercising	11.6	14.6	0.00	19.0	22.3	0.18

**Table 3:** Unstandardized multiple linear regression coefficients for contribution of age and gender to the model of the association between time of testing and mean HPLP-II scores for diet and physical activity.

	Adolescents (*n* = 3278)				Adults (*n* = 544)			
	β	95% CI	SE	*p*	β	95% CI	SE	*p*
*Diet*								
Age	−0.026	−0.030 to −0.022	0.002	<0.001	0.003	0.001 to 0.004	0.001	<0.001
Gender	−0.007	−0.030 to −0.017	0.012	0.579	0.027	−0.029 to 0.083	0.028	0.341
*Physical activity*								
Age	−0.030	−0.035 to −0.025	0.003	<0.001	0.004	0.002 to 0.006	0.001	<0.001
Gender	−0.079	−0.109 to −0.050	0.015	<0.001	−0.082	−0.164 to 0.001	0.042	0.053

CI—confidence intervals, SE—standard error of estimate.
